# Photonic neuromorphic architecture for tens-of-task lifelong learning

**DOI:** 10.1038/s41377-024-01395-4

**Published:** 2024-02-26

**Authors:** Yuan Cheng, Jianing Zhang, Tiankuang Zhou, Yuyan Wang, Zhihao Xu, Xiaoyun Yuan, Lu Fang

**Affiliations:** 1 Sigma Laboratory, Department of Electronic Engineering, Tsinghua University, Beijing, 100084 China; 2 Beijing National Research Center for Information Science and Technology (BNRist), Beijing, 100084 China; 3 Institute for Brain and Cognitive Science, Tsinghua University (THUIBCS), Beijing, 100084 China

**Keywords:** Photonic devices, Applied optics

## Abstract

Scalable, high-capacity, and low-power computing architecture is the primary assurance for increasingly manifold and large-scale machine learning tasks. Traditional electronic artificial agents by conventional power-hungry processors have faced the issues of energy and scaling walls, hindering them from the sustainable performance improvement and iterative multi-task learning. Referring to another modality of light, photonic computing has been progressively applied in high-efficient neuromorphic systems. Here, we innovate a reconfigurable lifelong-learning optical neural network (L^2^ONN), for highly-integrated tens-of-task machine intelligence with elaborated algorithm-hardware co-design. Benefiting from the inherent sparsity and parallelism in massive photonic connections, L^2^ONN learns each single task by adaptively activating sparse photonic neuron connections in the coherent light field, while incrementally acquiring expertise on various tasks by gradually enlarging the activation. The multi-task optical features are parallelly processed by multi-spectrum representations allocated with different wavelengths. Extensive evaluations on free-space and on-chip architectures confirm that for the first time, L^2^ONN avoided the catastrophic forgetting issue of photonic computing, owning versatile skills on challenging tens-of-tasks (vision classification, voice recognition, medical diagnosis, etc.) with a single model. Particularly, L^2^ONN achieves more than an order of magnitude higher efficiency than the representative electronic artificial neural networks, and 14× larger capacity than existing optical neural networks while maintaining competitive performance on each individual task. The proposed photonic neuromorphic architecture points out a new form of lifelong learning scheme, permitting terminal/edge AI systems with light-speed efficiency and unprecedented scalability.

## Abstract (web summary)

Inspired by human brain for multi-task continual learning, a generalized photonic neuromorphic architecture (L^2^ONN) is proposed to model physical-driven light sparsity and parallelism, towards reconfigurable and scalable lifelong learning.

## Graphical abstract

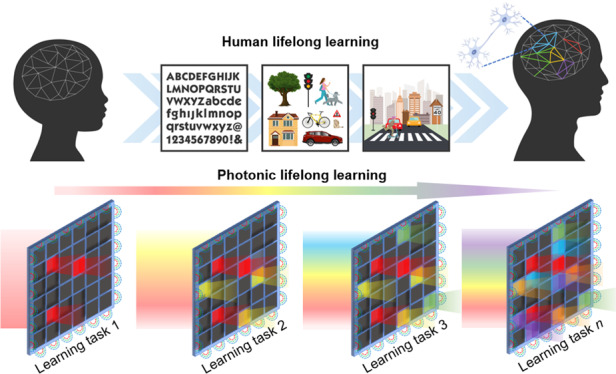

## Introduction

Artificial intelligence (AI) tasks become increasingly abundant and complex fueled by large-scale datasets^1–4^. One open question in the field of machine learning is how artificial agents could propagate in a smarter manner with exceptional learning scalability and realize versatile advanced AI tasks^5–8^. With the plateau of Moore’s law and end of Dennard scaling, energy consumption becomes a major barrier to more widespread applications of today’s heavy electronic deep neural models^9–12^, especially in terminal/edge systems^13,14^. The community is imminently looking for next-generation computing modalities to break through the physical constraints of electronics-based implementations of artificial neural networks (ANNs).

Photonic computing has been promised to overcome the inherent limitations of electronics and improve energy efficiency, processing speed and computational throughput by orders of magnitude^15–17^. Such extraordinary properties have been exploited to construct application-specific optical architectures^18–22^ for solving fundamental mathematical and signal processing problems with performances far beyond those of existing electronic processors. Optical neural networks (ONNs) are constructed to validate simple visual processing tasks^23–26^ such as hand-written digit recognition^27–29^ and saliency detection^30,31^, using wave-optics simulations or small-scale photonic computing systems. Meanwhile, some works combine the photonic computing units with a variety of electronic ANNs to enhance the scale and flexibility of optical architectures, e.g., deep optics^32–34^, amplitude-only Fourier ONNs^31^, and hybrid optical-electronic CNN^35^. However, existing optics-based implementations are limited to a small range of applications and cannot continually acquire versatile expertise on multiple tasks to adapt to new environments. The main reason is that they inherit the widespread problem of conventional computing systems, which are prone to train new models interfering with formerly learned knowledges, rapidly forget the expertise on previously learned tasks when trained on something new, i.e., ‘catastrophic forgetting’^36–40^. Such an approach fails to fully exploit the intrinsic properties in sparsity and parallelism of wave optics for photonic computing, which ultimately results in poor network capacity and scalability for multi-task learning.

In contrast, humans possess the unique ability to incrementally absorb, learn and memorize knowledge. In particular, neurons and synapses perform work only when there are tasks to deal with, in which two important mechanisms participate: sparse neuron connectivity^41–43^ and parallelly task-driven neurocognition^44–47^, together contribute to a lifelong memory consolidation and retrieval. Accordingly, in ONNs, these characteristic features can be naturally promoted from biological neurons to photonic neurons based on the intrinsic sparsity and parallelism properties of optical operators^31,48–51^. An optical architecture imitating the structure and function of human brains demonstrates its potential to alleviate the aforementioned issues, which shows more advantages than electronic approaches in constructing a viable lifelong learning computing system.

**Fig. 1:**
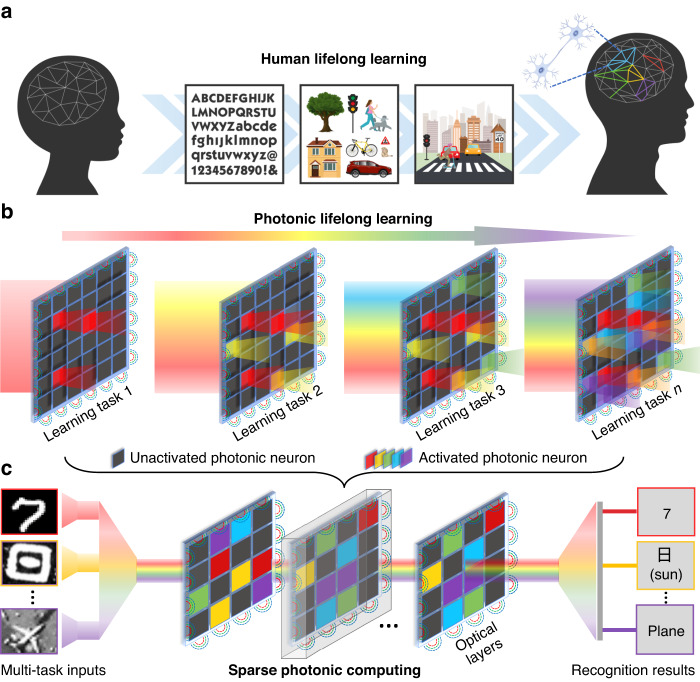
Principle of the photonic neuromorphic architecture. **a** Illustration of human lifelong learning. The brain can incrementally absorb, learn and memorize knowledge throughout its lifespan. Neurons and synapses are adaptively connected by task-driven neurocognition. **b** Diagram of the neuromorphic photonic lifelong learning. The photonic connections in each optical layer are gradually activated with different tasks. Photonic neurons only lighten when activated by corresponding signals, in which the active connections are relatively sparse and the information is parallelly transmitted in spectrum. **c** Workflow of the L^2^ONN multi-task inference. Input information of multiple tasks is encoded into coherent light with different wavelengths, and processed with the sparse photonic computing module to obtain the final results

**Fig. 2:**
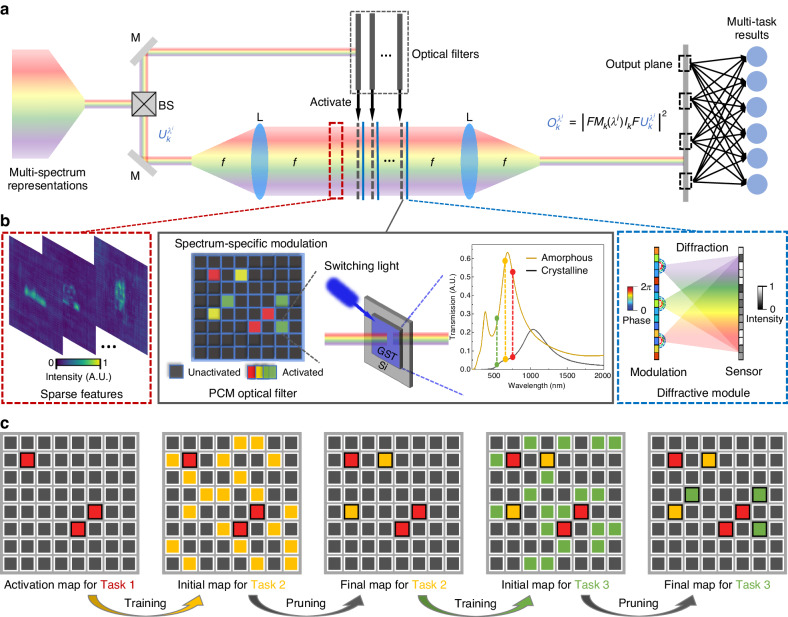
Free-space implementation of photonic lifelong learning (L^2^ONN). **a** Overall structure of L^2^ONN. Inputs of multi-tasks are projected into coherent light field with the multi-spectrum representations ${U}_{k}^{{\lambda }^{i}}$. Beam splitter (BS), mirrors (M), lens (L) and optical filters are employed to guide and modulate the light. The cascaded sparse optical layers are realized by configuring the light-controlled optical filters at the Fourier plane of a 4$f$ optical system. With propagation of optical feature embeddings *O* at the output plane, the final results can be obtained through an electronic read-out layer. **b** Detailed construction of the reconfigurable optical layers. Each layer receives sparse features as the inputs. PCM-based filters are all-optically switched, which sparsely conducts spatial and spectrum-wise photonic neuron activations. The activated photonic neurons are then connected in the subsequent diffractive computing module. **c** Training strategy of photonic lifelong learning on an 8 × 8 optical filter. Training of each task initially learns a dense activation map, which is further pruned to a sparse one. The activation map of each task is retained and stay fixed in the following evolution of learning. The final filter shares optical weights learned from all seen tasks

Herein, we propose L^2^ONN: a reconfigurable photonic computing architecture for lifelong learning (Fig. 1). Neuromorphically inspired, L^2^ONN can incrementally learn tens-of-tasks in one model with light-speed efficient computation. We show that the unique characteristics of light, spatial sparsity and multi-spectrum parallelism that for the first time developed in photonic computing architecture, endow ONNs with lifelong learning capability. Specifically, considering the physical propagation of free-space coherent light field (Fig. 2): Phase change materials (PCM)-based sparse optical filters are employed to modulate photonic neuron connections of each single task; And a multi-spectrum light diffraction-based optical computing module is constructed to extract the multi-task features allocated with different wavelengths. Throughout the architecture, photonic neurons are selectively activated according to the input signals. Unlike existing ONNs trying to imitate ANN structures, the photonic lifelong learning of L^2^ONN is initially designed following the physical nature of light-matter interaction, to fully explore the functional and performance potentials of wave optics in photonic computing.

**Fig. 3:**
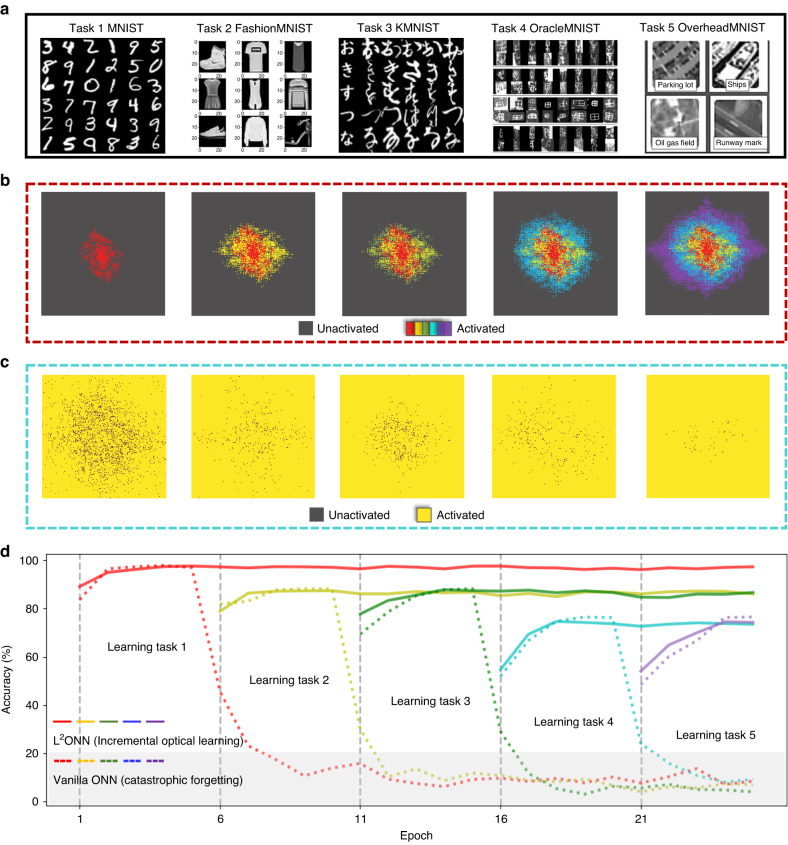
Evaluation on the photonic lifelong learning capability. **a** 5 representative vision classification tasks used for training L^2^ONN. Evolution of the activation map in layer 1 of **b** L^2^ONN and **c** vanilla ONN. With task learning, the photonic neuron connections in L^2^ONN are initially sparse and constantly enlarged, colored with red, yellow, green, blue and purple, respectively, while in vanilla ONN are quite dense from the first task. **d** Convergence comparison between L^2^ONN and vanilla ONN. Each task is trained for 5 epochs, L^2^ONN can increment its capabilities and memorize all seen tasks, while vanilla ONN rapidly forgets what was learned before and falls into the catastrophic forgetting area (below 20% accuracy)

**Fig. 4:**
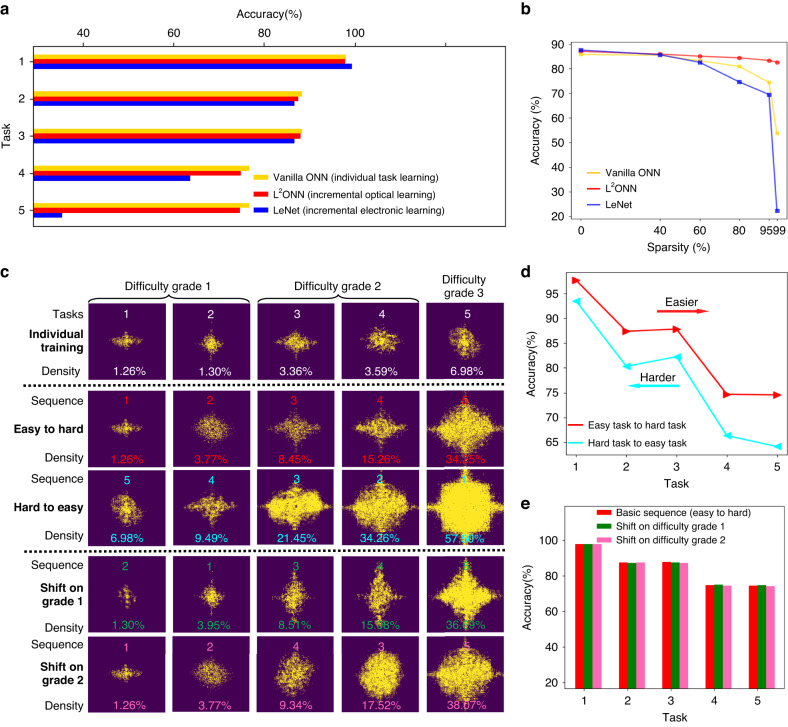
Numerical performance of photonic lifelong learning. **a** Accuracy comparison among different benchmarks of vanilla ONN, L^2^ONN and LeNet. The electronic approach LeNet is installed with similar pruning rate and incrementally learns tasks using the same training strategy. **b** Analysis on network sparsity with the individual FashionMNIST task. All approaches are configured with fixed pruning settings under the same sparsity. **c** Evolution of the proposed optical filters with various training sequences. 5 tasks are divided into 3 task difficulty grades according to the photonic neuron activation density of each individual training (row 1). Tasks 1 and 2, and tasks 3 and 4 share the same grade due to they have the similar densities. Based on such criteria, **d** training sequences of easy to hard and hard to easy (rows 2 and 3) are evaluated, and **e** shifts of interior sequences on grades 1 and 2 (rows 4 and 5) are further reported

The free-space L^2^ONN can adaptively allocate computational resources with unprecedented scalability and versatility, permitting ONNs to increment capabilities and memorize knowledges with enhanced performance. In the experiments, for the first time, we evaluate that L^2^ONN can progressively learn challenging tens-of-tasks, e.g., from hand-written digit classification to complex scene recognition (Fig. 3). The network achieves up to 14× larger learning capacity than the vanilla ONN^52^ while maintaining competitive accuracy on each individual task, and more than an order of magnitude higher efficiency than the representative electronic based neural networks, e.g., LeNet^53^. It is worth noting that the learning sequence on complexity of tasks affects much on overall network performance (Fig. 4). The smarter way is to start from an easy task and slowly transition to more difficult ones, which corresponds with the progressive learning styles of human.

**Fig. 5:**
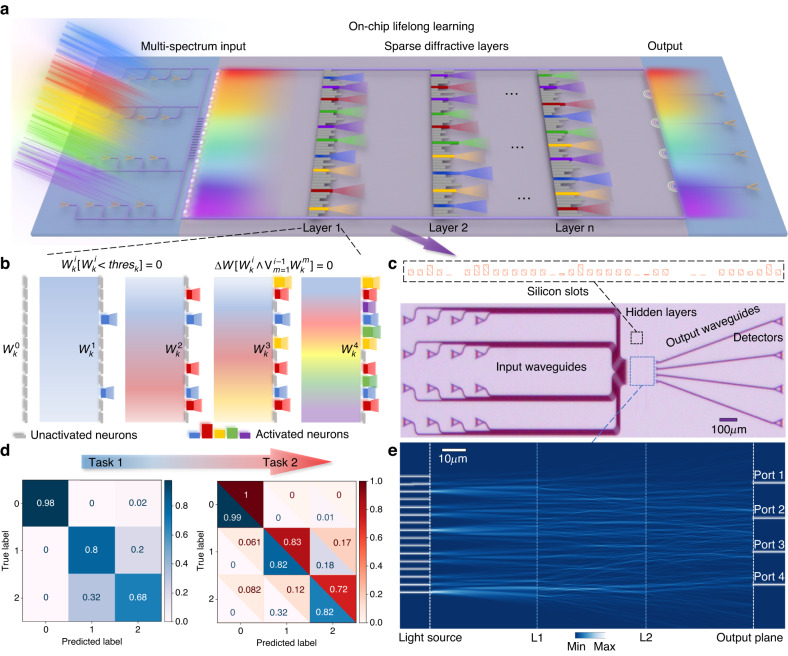
Photonic lifelong learning on chip. **a** Schematic of an on-chip L^2^ONN architecture. Each color represents a different task, multi-task inputs are encoded into optical signals and transmitted by multi-spectrum wave sources. The optical features are propagated with several sparse diffractive layers composed of silicon slots, each slot represents a single photonic neuron. **b** Evolution of a single layer during the multi-task learning process. **c** Micrograph of a real fabricated all-optical chip including 2 hidden layers, 16 input waveguides and 4 detectors. **d** Confusion matrices of the on-chip lifelong learning on 2 representative classification datasets. **e** The optical field propagation of L^2^ONN with FDTD that performs task 2 inference after learning both tasks

An on-chip L^2^ONN is designed and fabricated for further validation, which experimentally verifies its lifelong learning performance on representative classification tasks in an all-optical and scalable manner (Fig. 5). The chip can realize a low-cost mass manufacturing based on standard CMOS technology, it is promising to implement L^2^ONN as a photonic accelerator onto the highly-integrated terminal/edge AI systems. We expect that our study will provide a light-speed and low-power solution to practically tackle real-world manifold tasks, meanwhile breaking through the energy and scaling walls towards more extensive applications of transformative AI techniques.

## Results

Humans possess an extraordinary capacity to retain memories and increment new knowledges throughout their lifespan. The process of human lifelong learning is illustrated in Fig. 1a, the brain can progressively absorb, learn and memorize knowledges, e.g., evolving from recognizing basic characters and objects to understanding complex scenes. During learning, neurons and synapses are gradually activated and connected to remember specified tasks, which only function when there are task-related external stimuli. We depict that two important neurocognitive mechanisms participate here: sparse neuron connections and parallel task-driven processing, which can be naturally promoted from biological neurons to photonic neurons based on the intrinsic sparsity and parallelism of light.

Neuromorphically inspired, the principle of photonic lifelong learning is illustrated in Fig. 1b. Each stage activates a new set of photonic neurons represented with a new color. These updated neurons encode the newly learned knowledge, and will be consolidated to avoid catastrophic forgetting in future learning, just like human never forgets basic skills, e.g. how to ride a bicycle. Schematic of the proposed free-space L^2^ONN workflow of multi-task inference is presented in Fig. 1c. The inputs of multiple tasks are encoded into coherent light field with different wavelengths, and parallelly delivered into the cascaded sparse optical layers. Through light-wave propagation, the optical features are further processed and the inference results are calculated. The learning strategy and training method are shown in Fig. S1. Along with the photonic lifelong learning, L^2^ONN can obtain versatile expertise on challenging tens-of-tasks adapting to new scenarios, such as vision classification (Fig. 3), voice recognition (Fig. S6), and medical diagnosis (Fig. S7).

The free-space implementation of L^2^ONN architecture is proposed in Fig. 2. Specifically, Fig. 2a illustrates the overall structure, where the inputs are transferred into multi-spectrum representations bearing multi-task information, projected to a shared domain, and propagated through the diffraction computing module, which is cascaded by sparse optical layers in the Fourier plane of a coherent 4$f$ optical system^30^. Each layer consists of an optical filter which is adaptively switched in accordance with different tasks, and a diffractive unit modulates the subsequent light field. Thus, photonic neurons can be selectively activated dependent on input signals. Outputs of each layer will be remapped as inputs to next except last one. Final optical outputs will be detected on output plane and further fed into an electronic read-out layer for recognition results. Detailed layer size and depth of L^2^ONN are presented in Fig. S2.

Detailed construction of a single layer is presented in Fig. 2b. The layer receives originally sparse features from previous layer and performs optical diffraction for subsequent layers. Particularly, we adopt phase change materials (PCM)^54,55^ for optical filters to switch both spatial and spectrum-wise activations. The applied PCM is composed of GeSbTe (GST) growing on a transparent Si substrate. Each GST cell has 2 states of amorphous and crystalline with different transmission spectra, which can be switched instantly by the control light (see Fig. S4). The all-optical control ensures that the modulations on phase and intensity are conducted with minimal delay. Under a fixed wavelength, we define the GST cells with higher transmission as activated and lower transmission as unactivated. Such PCM-based spectrum-specific modulation realizes higher performance than the on-off binary modulation based on digital micromirror device (DMD) (see Fig. S3 and Table S4). Furthermore, the selection of wavelengths shows evident effects on the network performance. After investigation, that working wavelengths are configured with gap of 50 nm to achieve highest accuracy (see Fig. S9 and Table S4).

Figure 2c shows the multi-task training strategy of L^2^ONN using an 8 $\times$ 8 optical filter. The primitive states of all PCM cells stay unactivated and incrementally activate along with the training process. For each new task, the optical filter initially learns a dense activation map, which is further pruned to a sparse one utilizing an intensity threshold (details in Method), only the photonic neurons of intensity beyond threshold will be activated and keep fixed in the following evolution of learning. The activation map on filter shares optical weights learned from all seen tasks and gradually acquires versatile expertise on new tasks to adapt to new environments, avoiding the catastrophic forgetting issue of conventional ONNs.

The photonic lifelong learning capability (Fig. 3) and numerical performance (Fig. 4) of a three-layer free-space L^2^ONN (details in Fig. S2) is validated on 5 representative vision classification tasks^56–60^ in Fig. 3a. L^2^ONN is incrementally trained on these 5 tasks and the evolution on activation map of layer 1 is obtained in Fig. 3b, which gradually enlarges and remains fixed along with the following task learning. It can be observed that L^2^ONN only requires a fraction of photonic neuron activation to grasp each new task.

We contrastively construct a three-layer vanilla ONN with the same amount of parameters and also a computational equivalent five-layer electronic LeNet (see Fig. S2) incrementally learning tasks in the same way. Figure 3c shows the variation of photonic neuron activation map of vanilla ONN, which keeps dense during the whole training process. Each new task learning tends to fully occupy the parameter space and interfere with formerly learned ones, leading to the evident catastrophic forgetting issue. Figure 3d compares the convergence plots between L^2^ONN and vanilla ONN, 25 epochs are applied and 5 epochs for each task. Setting below 20% accuracy as the catastrophic forgetting baseline, vanilla ONN would rapidly experience the forgetting issue after 2 epochs of training new task, which indicates that the previously learned expertise has been almost erased. Differently, L^2^ONN can memorize the knowledges of all seen tasks and increment its capabilities on new tasks. Using a fixed activation threshold of 0.5, L^2^ONN can continually learn at most 14 tasks occupying totally 96.3% photonic neuron connections, while achieving more than an order of magnitude higher efficiency than the electronic ANN (see Note S1). Details about the dynamic evolution of activation map and accuracy variation are presented in Video S1. More evaluation results on vision classification are reported in Figs. S5, S8, Table S1. The proposed photonic lifelong learning architecture can adaptively allocate computational resources with unprecedented scalability, permitting ONN to acquire versatile expertise with superior learning capacity when dealing with continuous streams of new data.

Figure 4a reports the accuracy comparison among different benchmarks of vanilla ONN of individual task learning, L^2^ONN of incremental optical learning and electronic ANN of incremental electronic learning. The electronic ANN is installed with equivalent computations, applied with similar pruning rate and trained with the same training strategy as L^2^ONN. During the learning process, L^2^ONN with highly sparse photonic computing just loses at most 1.9% accuracy compared with the vanilla ONN with full connections, while only using 34.3% parameters of the vanilla ONN to grasp all 5 tasks. As for the comparison on incremental learning capability, the electronic ANN just gains a 1.2% accuracy improvement on the first task but gets lower accuracy on all rest of tasks when compared with L^2^ONN. More significantly, the electronic ANN suffers a rapid performance degradation from the 4-th task training, due to the lack of inherent sparsity compared with photonic computing (see Video S2).

Moreover, Fig. 4b compares the performance with different sparsity among vanilla ONN, L^2^ONN and electronic ANN on individual FashionMNIST task. The electronic ANN outperforms ONN-based approaches when the sparsity is below 40%, however, its performance visibly decreases if the sparsity is beyond 60%. In contrast, L^2^ONN robustly obtains competitive accuracy of 82.6% (only 3.1% reduced) when sparsity reaches 99% while vanilla ONN gets 53.8% and electronic ANN is 22.3%. In particular, L^2^ONN achieves 14× larger capacity than existing optical neural networks while maintaining competitive accuracy on each individual task. We conclude that optics own more instinct advantages in sparsity and parallelism than electronics due to the massive optical information, achieving equivalent or higher performance while costing fewer computational resources. More evaluations of L^2^ONN on voice recognition and medical diagnosis datasets are presented in Figs. S6, S7, S8 and Tables S2, S3.

Figure 4c investigates how learning sequence impacts the performance of photonic lifelong learning. First, we train L^2^ONN on each individual task with the same intensity threshold of optical filter and obtain the activation density of layer 1, which is regarded as the classifying criteria of task difficulty grade. Consequently, 5 tasks can be classified into 3 difficulty grades since tasks 1 and 2, and tasks 3 and 4 have similar densities. Under such standard, L^2^ONN is trained with 2 extreme training sequences of easy to hard and hard to easy, and their corresponding accuracy curves are compared in Fig. 4d. We observe that training from easy to hard costs less photonic neuron activation at all steps (23.25% at most) but achieves higher performance on all tasks (10.42% at most) when compared with the training from hard to easy. L^2^ONN further proves its human-like characteristics in lifelong learning which requires a step-by-step process to gradually absorb, memorize and consolidate skills, starting from complex tasks will receive the opposite effects, just like human always learns creeping before walking. Furthermore, we successively shift the interior sequences of difficulty grades 1 and 2 and report the evaluation results in Fig. 4e. Although spatial distributions show differences, the activation densities and accuracies barely vary from the basic training sequence (easy to hard).

The design and fabrication of the on-chip L^2^ONN architecture are depicted in Fig. 5. Figure 5a shows its holistic schematic. Multi-task inputs are encoded into optical signals and transmitted by multi-spectrum wave sources. The sparse diffractive layers are based on an integrated one-dimensional dielectric metasurface, which consists of a series of etched slots filled with silicon dioxide on device layer of silicon-on-insulator (SOI) substrate (see Fig. S10). Each slot functions as a single photonic neuron and acts as a secondary wave source, the amplitude and phase of which are determined by the product of the input wave and the complex-valued transmission at that neuron. During the sparse optical features propagating, neurons with lighted color represent activated by the corresponding tasks while the gray ones means unactivated.

As illustrated in Fig. 5b, the architecture conducts each task with a slot group and gradually enlarges the activations along with lifelong learning process. ${W}_{k}^{i}$ represent the activated neuron weights of $i$-th task in $k$-th layer, which are sparsely pruned utilizing an intensity threshold ${{thres}}_{k}$. The activation weights of each task are set fixed in the subsequent task training, while the unactivated neurons can be iteratively configured when new tasks are learned (details in Method). Figure 5c presents the micrograph of a real fabricated all-optical chip for photonic lifelong learning, which consists of a 16-channel data-input grating coupler array, a dual-layer diffractive modulation area and a 4-channel read-out grating coupler array (details in Note S2). Each hidden layer contains 1000 stand-alone slots corresponding to the diffractive photonic neurons. Specifically, the multi-task signals are fed into the sparse diffractive unit with 16 input waveguides, output intensity signals are measured by 4 detectors after modulation. The whole chip merely encompasses an area of under $1{{mm}}^{2}$, indicating high level of compactness and integration.

Figure 5d reports the confusion matrices along with the on-chip lifelong learning process on 2 representative datasets (Iris flower classifier^61^ and Red wine quality^62^). The datasets are transferred onto the phase of light and then used to train the sparse weights of diffractive unit. It can be observed that the proposed on-chip L^2^ONN can effectively avoid catastrophic forgetting issue and increment its experiences on new task. After training, the sparsely activated neurons are etched on slots to implement 2 tasks on a single chip. The optical field propagation using photonic finite-difference time-domain (FDTD) evaluation is shown in Fig. 5e, running a testing example from task 2. The amplitude of input light source mode in input ports represents data features while the light intensity detected with output plane delivers classification results. More details about multi-task training and FDTD analysis are shown in Figs. S11, S12. Experimental evaluation has verified that the proposed photonic chip can execute both tasks in an all-optical and scalable manner. It is promising to integrate the photonic lifelong learning mechanism into optoelectronic AI systems by replacing the off-the-shelf devices with on-chip L^2^ONN.

## Discussion

This paper innovates a reconfigurable photonic neuromorphic architecture for scalable tens-of-task lifelong learning (L^2^ONN). It learns each single task by adaptively activating sparse photonic neuron connections, while continually acquiring expertise on various tasks by gradually enlarging the photonic activation, multi-task optical features are parallelly processed by multi-spectrum representations allocated with different wavelengths. An on-chip L^2^ONN is fabricated and experimentally verified its lifelong learning performance by incrementally implementing tasks on a single chip.

Mechanism of the photonic lifelong learning is inspired by the fact of brain functions of protecting memories and accommodating new knowledges by leveraging sparse neuron connections and parallel task-driven neurocognition. Optics own more inherent advantages in sparsity and parallelism than electronic computing systems due to the massive optical information. Unlike the existing artificial intelligence methods are prone to train new models interfering with formerly learned knowledges, the proposed photonic neuromorphic architecture increments capabilities on multiple tasks and avoids the catastrophic forgetting issue. With the speed of light, L^2^ONN gains high capacity to continually acquire versatile expertise when confronted with continuous streams of new data.

In summary, we have demonstrated the photonic lifelong learning provides a turnkey solution for large-scale real-life AI applications with unprecedented scalability and versatility. L^2^ONN shows its extraordinary learning capability on challenging tens-of-tasks, such as vision classification, voice recognition and medical diagnosis, supporting various new environments. We anticipate that the proposed neuromorphic architecture will accelerate the development of more powerful photonic computing as critical support for modern advanced machine intelligence and towards beginning a new era of AI.

## Materials and methods

### Free-space architecture design

As shown in Fig. 2b, the proposed free-space L^2^ONN architecture is designed with a sparse diffractive computing module for light propagation and an electronic fully-connected layer for recognition result read-out. Specifically, the diffractive computing part is cascaded by several 200$\times$200 optical layers and formed into the Fourier plane of a 4$f$ optical system under coherent light. Beam splitter (BS), mirrors (M), lens (L) and PCM-based optical filters are employed to guide and modulate the photonic neuron connections, phase modulators are applied to extract and propagate optical features, and an optical intensity sensor is used at the output plane to capture the final results. Utilizing a multi-spectrum coherent light source, multi-task inputs are transferred into optical representations, projected to a shared domain, and propagated by light diffraction.

Assuming ${U}_{k}^{{\lambda }^{i}}$ is the input complex light field of $k$-th optical layer on allocated wavelength ${\lambda }^{i}$ of $i$-th learned task, a 2$f$ system under coherent illumination is adopted and ${U}_{k}^{{\lambda }^{i}}$ is Fourier transformed into:

1 $${U^{\prime} }_{k}^{{\lambda }^{i}}=F{U}_{k}^{{\lambda }^{i}}$$

where ${{U^\prime}}_{k}^{{\lambda }^{i}}$ represents the optical features in Fourier domain and $F$ denotes the Fourier transform matrix. ${{U^\prime}}_{k}^{{\lambda }^{i}}$ is further modulated by optical filter:

2 $${U^{\prime\prime} }_{k}^{{\lambda }^{i}}={I}_{k}{\left({\lambda }^{i}\right){M}_{k}U^{\prime} }_{k}^{{\lambda }^{i}}$$

where ${U^{\prime\prime} }_{k}^{{\lambda }^{i}}$ is the features after modulation, ${M}_{k}$ denotes the functions of phase and ${I}_{k}({\lambda }^{i})$ denotes the intensity modulation, which can adaptively prune and conduct the photonic neuron connections to enable various tasks. Later, ${U^{\prime\prime} }_{k}^{{\lambda }^{i}}$ is Fourier transformed back to the real space applying another 2$f$ system, whose normalized output of this layer ${O}_{k}^{{\lambda }^{i}}$ is measured by an intensity sensor:

3 $${O}_{k}^{{\lambda }^{i}}={{\rm{|}}F{U^{\prime\prime} }_{k}^{{\lambda }^{i}}{\rm{|}}}^{2}$$

Note that except for the last layer, we remap the output intensity of each layer to complex optical field as the input of the next layer:

4 $${U}_{k+1}^{{\lambda }^{i}}={remap}\left({O}_{k}^{{\lambda }^{i}}\right)$$

where ${remap}()$ function applies the corresponding nonlinearity to the photonic computing. Define the number of total layers as $n$ (set as 3 in our experiments), the final outputs of the sparse diffractive computing module ${O}_{n}^{{\lambda }^{i}}$ will be directly detected on output plane and spatially cropped into 14 $\times$ 14 blocks, and the intensity of each block is measured with sensor and fed into a 196 $\times$10 electronic fully-connected layer to obtain the final recognition results (see Fig. 1).

### Optical modeling and training

The L^2^ONN free-space and on-chip implementations consist of four basic units: propagation, phase modulator, sensor, and remapping. These units construct the reconfigurable optical layer. Diffraction propagation unit is formulated by the angular spectrum method, where zero paddings are further adopted to ensure the boundary condition of optical feature propagation. Phase modulator unit applies phase shifts to the input optical field. Sensor unit transfers the complex optical information of amplitude and phase to intensity. The intensity to pixel value mapping is linear due to the gamma correction set as 1. Remapping unit converts the normalized intensity back to complex optical field as inputs for the following layers. Here we adopt the remapping method from MONET^21^.

During training, the loss function is defined as:

5 $$L={L}_{{CEN}}\left({P}^{i},{G}^{i}\right)+\alpha \mathop{\sum }\limits_{k=1}^{n}({{\rm{||}}{I}_{k}({\lambda }^{i}){\rm{||}}}^{2}+{{\rm{||}}{M}_{k}{\rm{||}}}^{2})$$

where ${L}_{{CEN}}$ represents the softmax cross-entropy loss^63^, ${P}^{i}$ and ${G}^{i}$ are the network precision and ground truth of $i$-th task, and $\alpha$ denotes the normalization coefficient, respectively.

Illustration of training strategy is shown in Figs. 2c, 5b. We apply the intensity mask measured by sensor unit as photonic neuron activation map. For each new task, the optical filter initially learns a dense activation map, which is further pruned to a sparse one utilizing an intensity threshold:

6 $${{map}}_{k}^{i}[{{map}}_{k}^{i} < {{thres}}_{k}]=0$$

where ${{map}}_{k}^{i}$ denotes the trained map of *k*-th layer on $i$-th task. The key factor ${{thres}}_{k}$ is determined by training process of each layer on each task. In other word, the sparsity proportions of optical filters are also trained as hyperparameters across all layers to achieve best performance. Only the photonic neurons of intensity beyond threshold will remain activated and keep fixed in the following evolution of learning:

7 $$\Delta W\left[{{map}}_{k}^{i}\wedge {{{\bigvee }}}_{m=1}^{i-1}{{map}}_{k}^{m}\right]=0$$

where $\Delta W$ denotes the gradient matrix of backpropagation on optical weights $W$, operation $\bigwedge$ searches the indices of coincident cells between new and former maps, and operation $\bigvee$ gradually merges the photonic neurons on activation maps of all trained tasks.

The network model is implemented with PyTorch V1.11 running on a single NVIDIA RTX3090 graphic card. Network parameters are optimized using the Adam optimizer^64^. All benchmarks including vanilla ONN and LeNet for comparison are made under the same hardware and software environments.

### Dataset preparation

We use 5 representative machine vision datasets including MNIST^56^, FashionMNIST^57^, KMNIST^60^, OracleMNIST^58^ and OverheadMNIST^59^ for evaluation on the free-space L^2^ONN, and 2 typical classification datasets of Iris flower classifier^61^ and Red wine quality^62^ for implementation of on-chip L^2^ONN. Among them, MNIST is the classic handwritten digit classification dataset of 10 classes; Fashion-MNIST consists of 10 classes with fashion article images; KMNIST is a drop-in replacement for MNIST dataset with 10 classes in Japanese; OracleMNIST includes ancient Chinese characters from 10 categories; OverheadMNIST is a benchmark satellite dataset with overhead views of 10 important object; Iris flower classifier contains 3 classes where each class refers to a type of iris plant; and Red wine quality includes 3 classes of wine qualities.

In Figs. S6, S8, we also evaluate the free-space L^2^ONN on 6 voice recognition tasks with recognition patterns from Vowel, Number, Word, Command, Gender and UrbanSound. Vowel^65^ consists of 12 audio classes of Japanese vowels; Number, Word and Command come from subsets of Speech Commands^66^, which is a large-scale audio dataset of rich spoken words, these 3 subsets contain 10, 15, and 10 categories, respectively; Gender^67^ includes 4 classes of audios from male, female, boy and girl; UrbanSound^68^ collects 10 classes of urban sounds from Gun Shot, Dog bark, etc. To uniform the input format, the original voice data is preprocessed into mel-scale frequency cepstral coefficients (MFCC)^69^ with a pre-emphasis factor of 0.97.

In addition, free-space L^2^ONN is tested on 4 medical diagnosis datasets. As shown in Figs. S7, S8, BloodMNIST of 8 classes, OrganMNIST of 11 classes, PathMNIST of 9 classes and TissueMNIST of 8 classes are adopted for network evaluation. These datasets are all from subsets of MedMNIST^70^, which is a large-scale MNIST-like collection of standardized biomedical images.

### Supplementary information

Visual comparison on the evolution of L^2^ONN and vanilla ONN

Visualization of network sparsity and learning capacity

Supplementary information for photonic neuromorphic architecture for tens-of-task lifelong learning
