# Supplementary material for: Photonic neuromorphic architecture for tens-of-task lifelong learning
Source: Light Sci Appl. 2024 Feb 26;13:56. doi: 10.1038/s41377-024-01395-4 (PMC10894876; doi:10.1038/s41377-024-01395-4)
Supplement: Supplementary file 3 — Supplementary information for photonic neuromorphic architecture for tens-of-task lifelong learning [file 41377_2024_1395_MOESM3_ESM.docx]

**Supplementary Information for**

**Photonic neuromorphic architecture for tens-of-task lifelong learning**

Yuan Cheng^1,2,†^, Jianing Zhang^1,2,†^, Tiankuang Zhou^1,2^, Yuyan Wang^2^, Zhihao Xu^1^, Xiaoyun Yuan^1,3^, Lu Fang^1,2,3,*^

^1^ Sigma Laboratory, Department of Electronic Engineering, Tsinghua University, Beijing, China.

^2^ Beijing National Research Center for Information Science and Technology (BNRist), Beijing, China.

3 Institute for Brain and Cognitive Science, Tsinghua University (THUIBCS), Beijing, China.

^†^ These authors contributed equally to this work.

* Corresponding author. Email: fanglu@tsinghua.edu.cn.

**Outline**

**Fig. S1.** System learning flowchart of the L^2^ONN.

**Fig. S2.** Detailed frameworks of benchmark models.

**Fig. S2.** Evaluation on optical filters in the free-space L^2^ONN.

**Fig. S4.** Illustration of programming process to amorphize and crystallize the integrated PCM cell.

**Fig. S5.** The confusion matrices of free-space L^2^ONN on 5 representative vision classification tasks.

**Fig. S6.** Photonic lifelong learning of free-space L^2^ONN on voice recognition.

**Fig. S7.** Photonic lifelong learning of free-space L^2^ONN on medical diagnosis.

**Fig. S8.** Comparisons among free-space L^2^ONN, vanilla ONN on individual task and vanilla ONN learning all tasks.

**Fig. S9.** Evaluation on multi-spectrum representation settings.

**Fig. S10.** Schematic of the on-chip L^2^ONN structure.

**Fig. S11.** Training process of on-chip L^2^ONN on 2 representative classification tasks.

**Fig. S12.** FDTD evaluations of on-chip L^2^ONN along with the lifelong learning process.

**Table S1.** Accuracy summarization of different benchmarks on 5 representative vision classification tasks.

**Table S2.** Accuracy summarization of different benchmarks on 6 voice recognition tasks.

**Table S3.** Accuracy summarization of different benchmarks on 4 medical diagnosis tasks.

**Table S4.** Ablation analysis of accuracy on optical filter and multi-spectrum representation.

**Note S1.** Computing efficiency analysis.

**Note S2.** On-chip fabrication.

**Video S1.** Visual comparison on the evolution of L^2^ONN and vanilla ONN.

**Video S2.** Visualization of network sparsity and learning capacity.


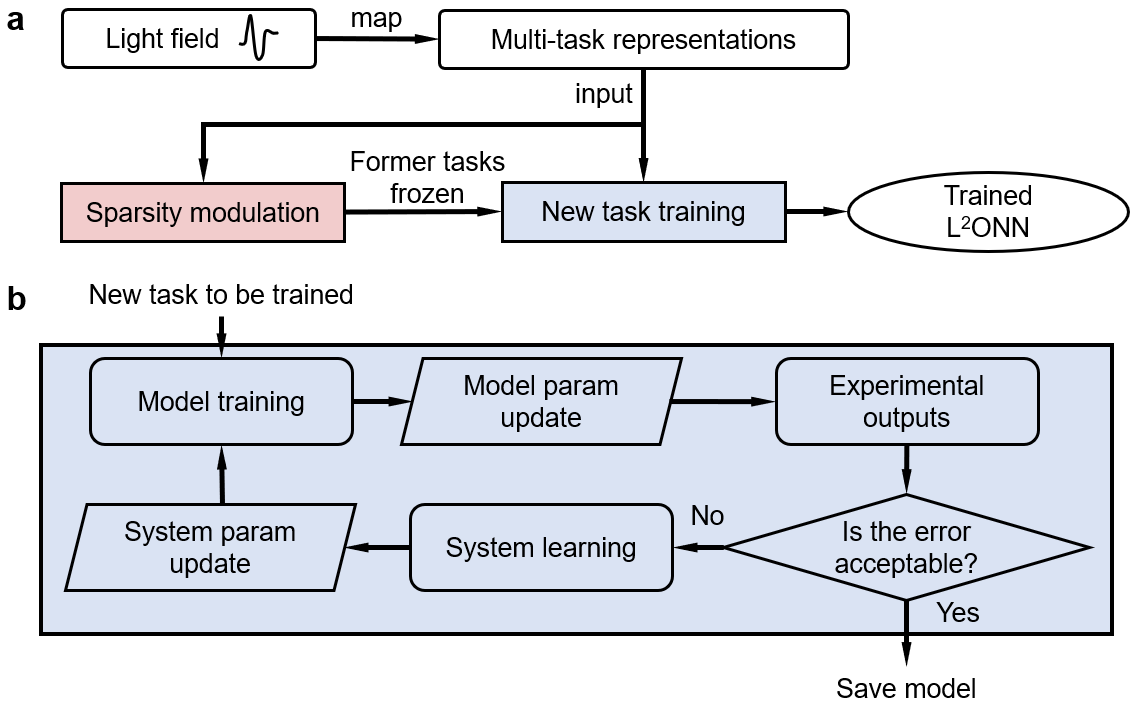


**Fig. S1: System learning flowchart of the L^2^ONN.** **a**, Lifelong learning strategy and **b**, training method. The system is simulated and trained on an electronic computer with configured calibration. The proposed adaptive training approach can overcome model deviation and restore inference accuracy by iteratively fine-tuning the network parameters layer by layer. The process of system learning and network training is iterated until the error decreases to an acceptable level.


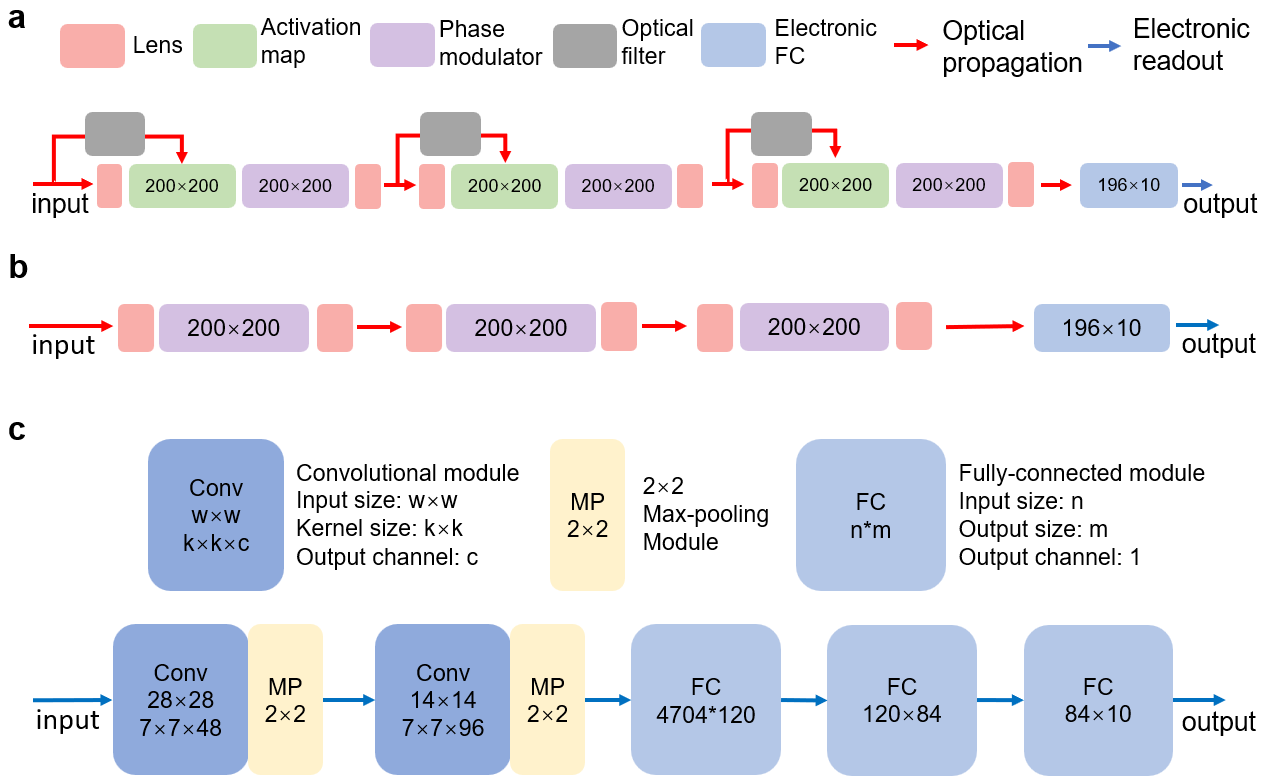


**Fig. S2: Detailed frameworks of benchmark models. a**, Structure of the proposed free-space L^2^ONN used in evaluations. Generally, it contains 3 sparse optical convolutional layers for light propagation, each optical layer consists of a PCM-based filter, a photonic map and a phase modulator with 200×200 resolution. A 196×10 electronic fully-connected layer is further employed to read out the recognition results from optical features. **b**, Vanilla ONN used for comparison. Neither optical filter nor photonic map is applied, other parts are just the same as free-space L^2^ONN. **c**, Electronic neural network (LeNet-5) used for comparison. It is composed of 2 convolutional layers, 2 max-pooling layers and 3 fully-connected layers. The applied LeNet- 5 model has the similar number of parameters as L^2^ONN.


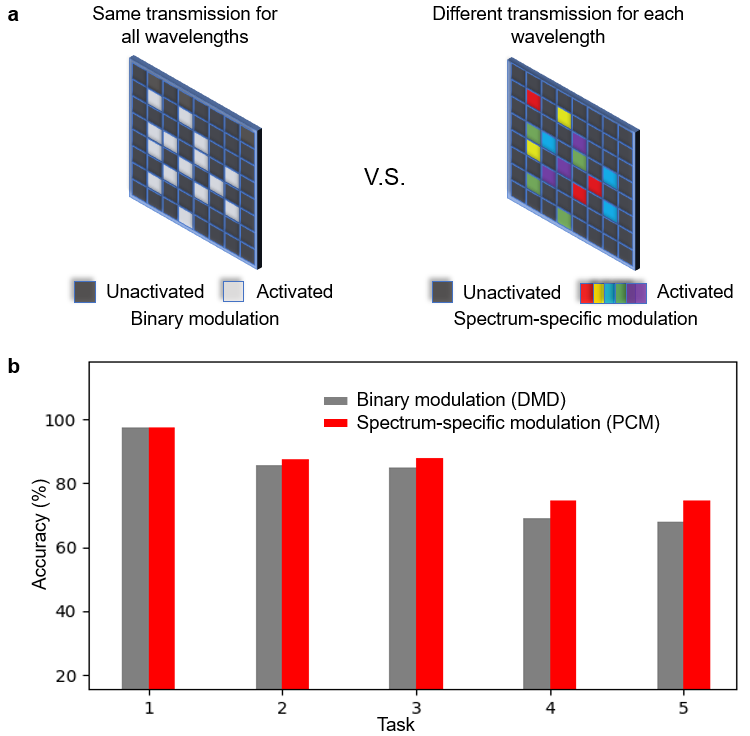


**Fig. S3: Evaluation on optical filters in the free-space L^2^ONN. a**, Differences between digital micromirror device (DMD) of binary modulation which presents the same transmission for all wavelengths, with phase change materials (PCM) of spectrum-specific modulation which performs different transmission for each adopted wavelength. **b**, Accuracy comparison between 2 kinds of optical filters of free-space L^2^ONN architecture on 5 representative vision classification tasks. The PCM-based optical filter outperforms DMD on all tasks, which demonstrates its unique benefits arising from spectral-wise modulation.


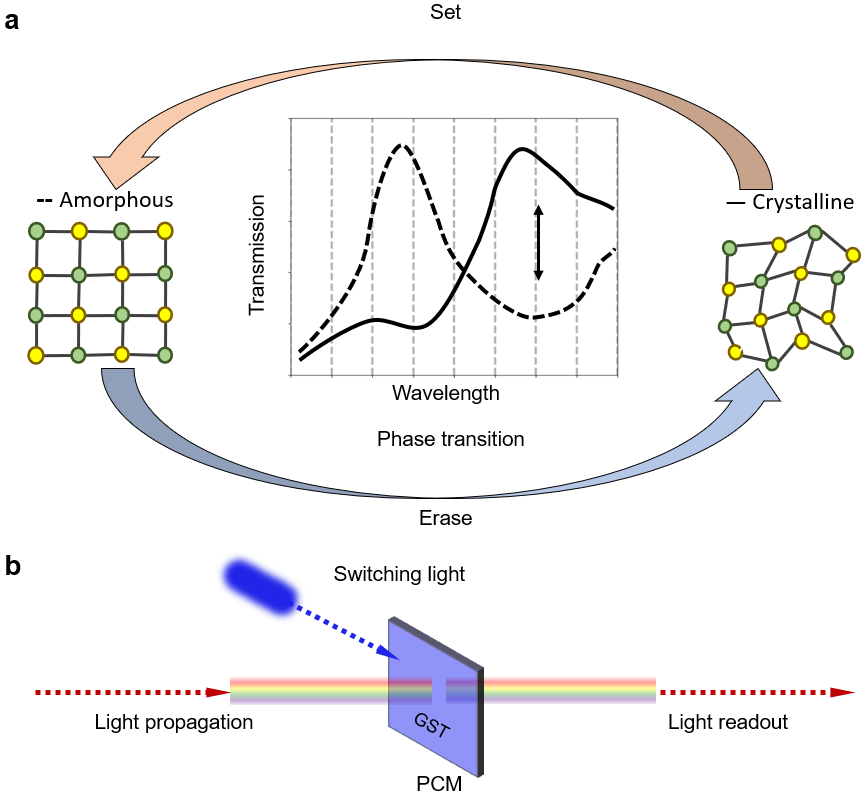


**Fig. S4: Illustration of programming process to amorphize and crystallize the integrated PCM cell. a**, Adaptive spectral-wise modulation is realized by switching different light transmissions with phase transition (set/erase) between crystalline and amorphous states of each PCM cell. **b**, Specifically, GeSbTe materials are adopted for PCM cells. Their states are transferred with an instant switching light with minimal delay.


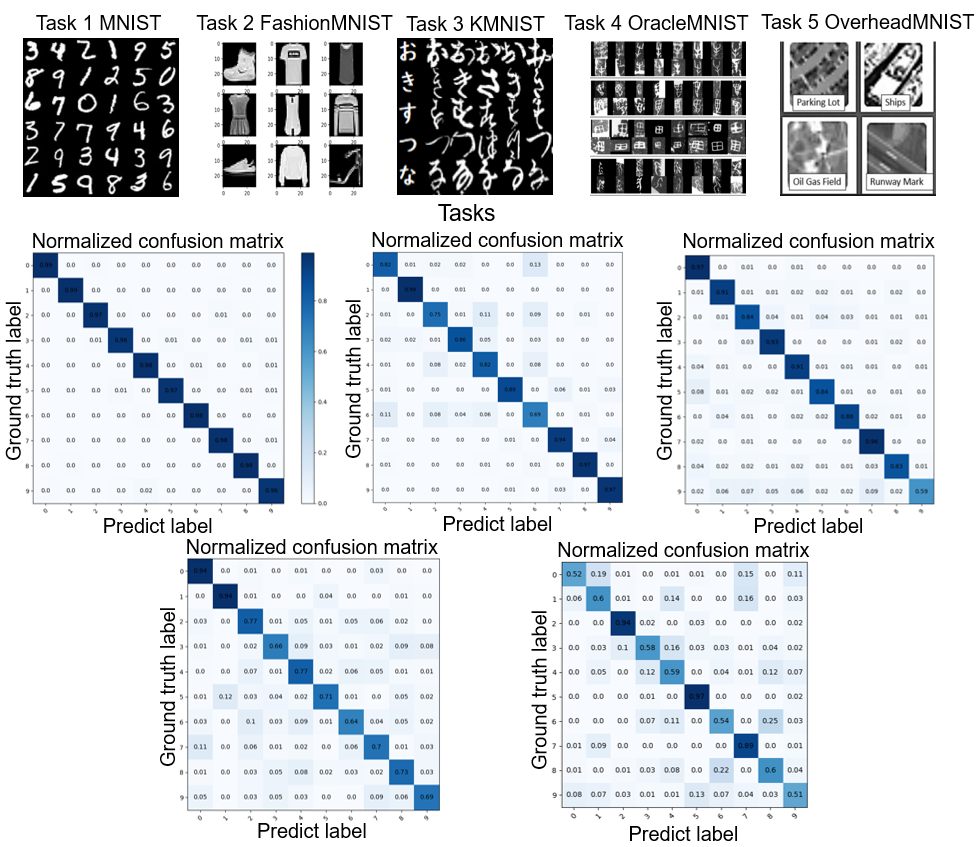


**Fig. S5:** **The confusion matrices of free-space L^2^ONN on 5 representative vision classification tasks.** We observe that the proposed L^2^ONN maintains competitive performance on all incrementally learned tasks.


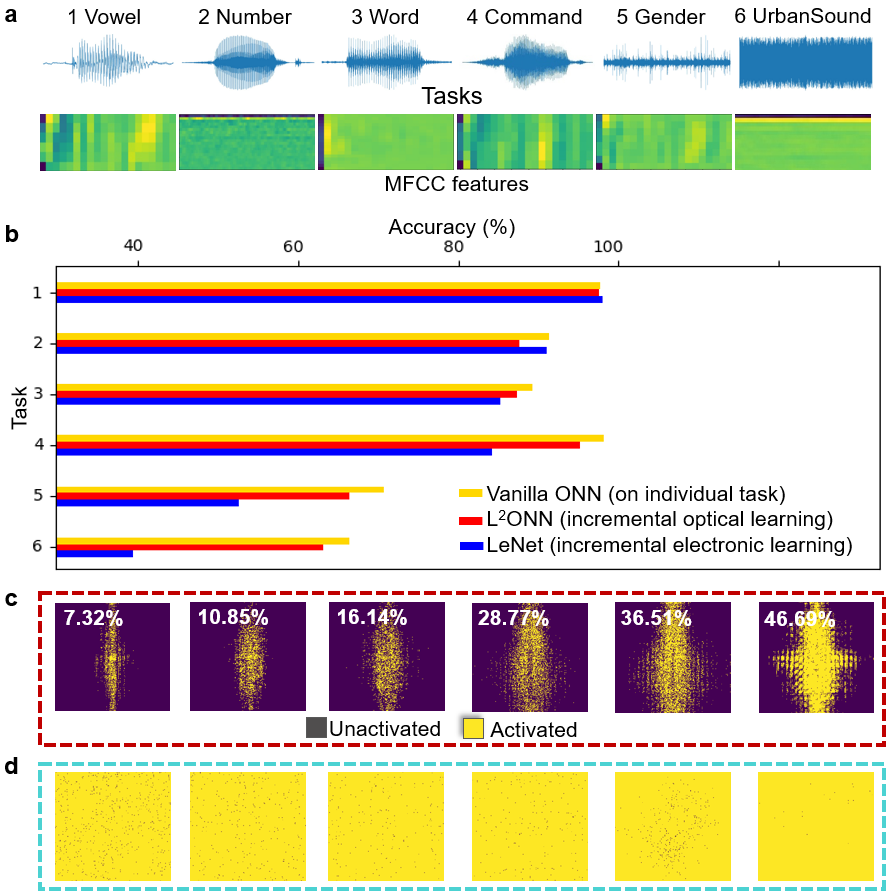


**Fig. S6: Photonic lifelong learning of free-space L^2^ONN on voice recognition. a**, 6 voice recognition datasets and samples of their corresponding mel-scale frequency cepstral coefficients (MFCC) transferred from the original voice data. **b**, Accuracy comparison among different benchmarks of vanilla ONN, L^2^ONN and LeNet. The electronic approach LeNet-5 is installed with initial 60% pruning and incrementally learns tasks using the same training strategy as L^2^ONN. Density of activated photonic neurons in layer 1 of **c**, L^2^ONN and **d**, vanilla ONN. With network learning, the photonic neuron connections in L^2^ONN are initially sparse and constantly activated, while in vanilla ONN are quite dense from the first task.


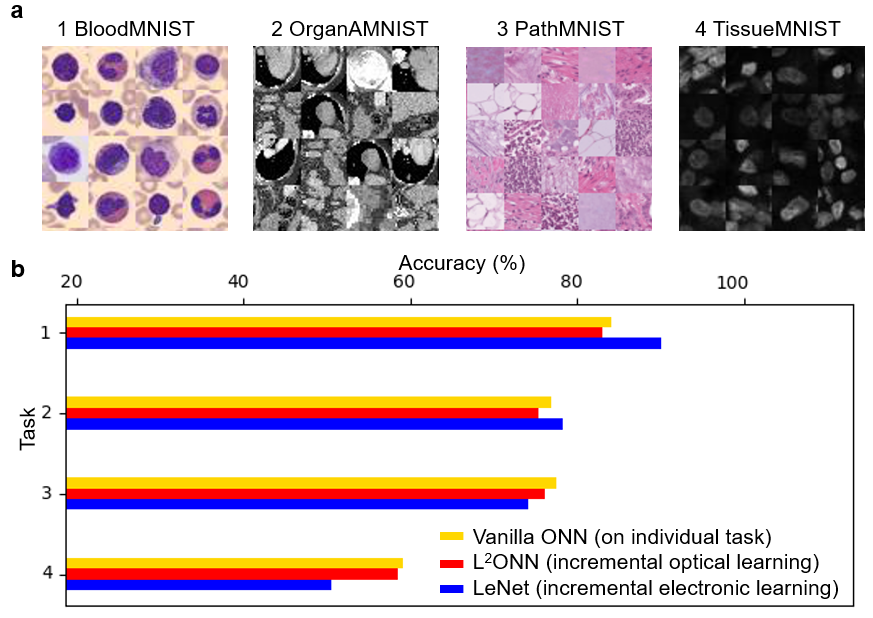


**Fig. S7: Photonic lifelong learning of free-space L^2^ONN on medical diagnosis. a**, 4 medical datasets used for lifelong learning. **b**, Accuracy comparison among different benchmarks of vanilla ONN, L^2^ONN and LeNet. The electronic approach LeNet-5 is installed with initial 40% pruning and incrementally learns tasks using the same training strategy as L^2^ONN.


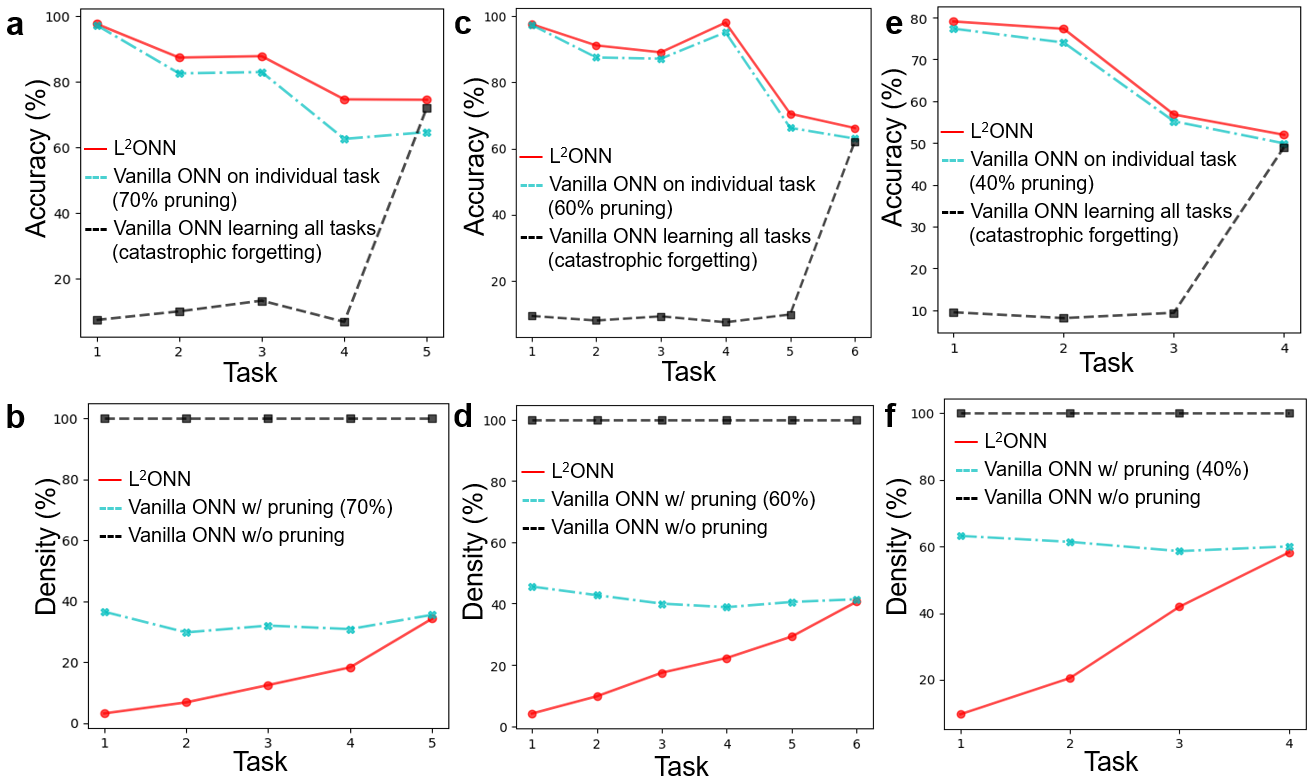


**Fig. S8: Comparisons among free-space L^2^ONN, vanilla ONN on individual task and vanilla ONN learning all tasks.** **a**, and **b**, report the accuracy and density on 5 representative vision classification tasks, respectively; **c**, and **d**, are on voice recognition tasks; and **e**, and **f**, are on medical diagnosis tasks. For intuitive comparison, the pruned vanilla ONN is initially set with same pruning rate as the minimum sparsity of L^2^ONN. One can observe that L^2^ONN achieves multi-task learning capability with higher accuracy on each task, while costs even fewer computational resources than vanilla ONN.


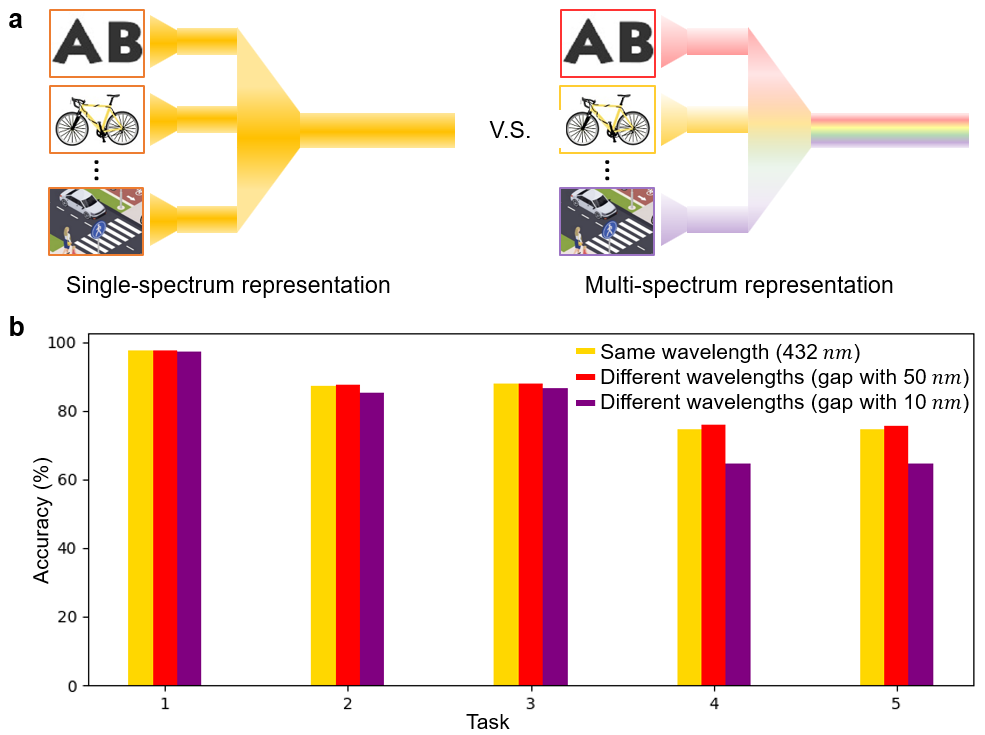


**Fig. S9: Evaluation on multi-spectrum representation settings. a,** Differences between single-spectrum representation of using same wavelength for all tasks, with multi-spectrum representation of allocating different wavelengths to every task. **b,** Accuracy comparison among various spectral settings of same wavelength, and different wavelengths with gaps of $50 nm$ and $10 nm$. We conclude that applying different wavelengths with gap of $50 nm$ is the best setting with highest performance for L^2^ONN.


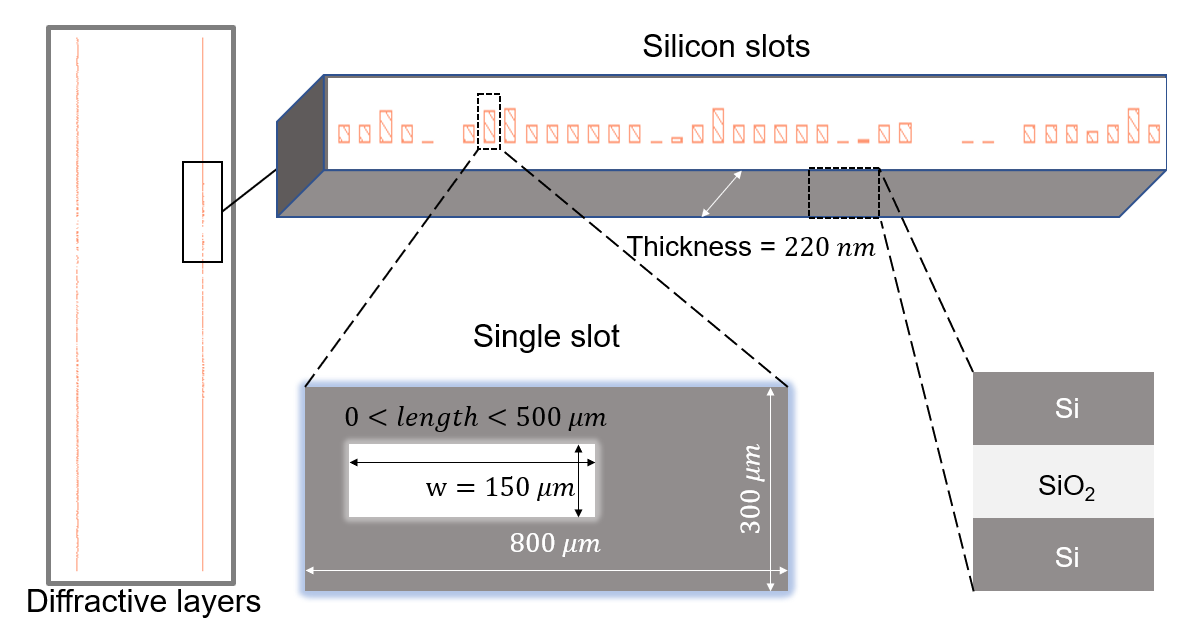


**Fig. S10: Schematic of the on-chip L^2^ONN structure.** The whole architecture contains 2 sparse diffractive computing layers. Each photonic neuron in layer consists of three identical silicon slots filled with silicon dioxide. The fabrication of on-chip L^2^ONN follows: $220 nm$ thickness of the silicon membrane; $300 \mu m$ distance of the adjacent slots; $150 \mu m$ width and $0\sim500 \mu m$ length of each slot; $8.5\sim11.5 \Omega$ of resistivity and $8\%$ of radial resistivity gradient; 3.89 of refraction index and 99.4% of transmission ratio (at $600 nm$ wavelength). These parameters were applied into experimental simulation to reversely design the proposed photonic architecture, the length of each slot is trained by deep learning method to represent a complex-valued transmission coefficient in L^2^ONN.


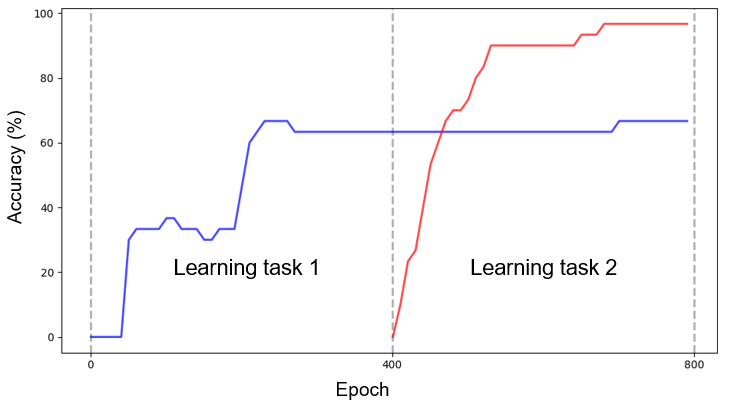


**Fig. S11: Training process of on-chip L^2^ONN on 2 representative classification tasks.** Each task is trained for 400 epochs. It can be observed that the proposed on-chip L^2^ONN can effectively avoid catastrophic forgetting issue and increment its experiences on new task.


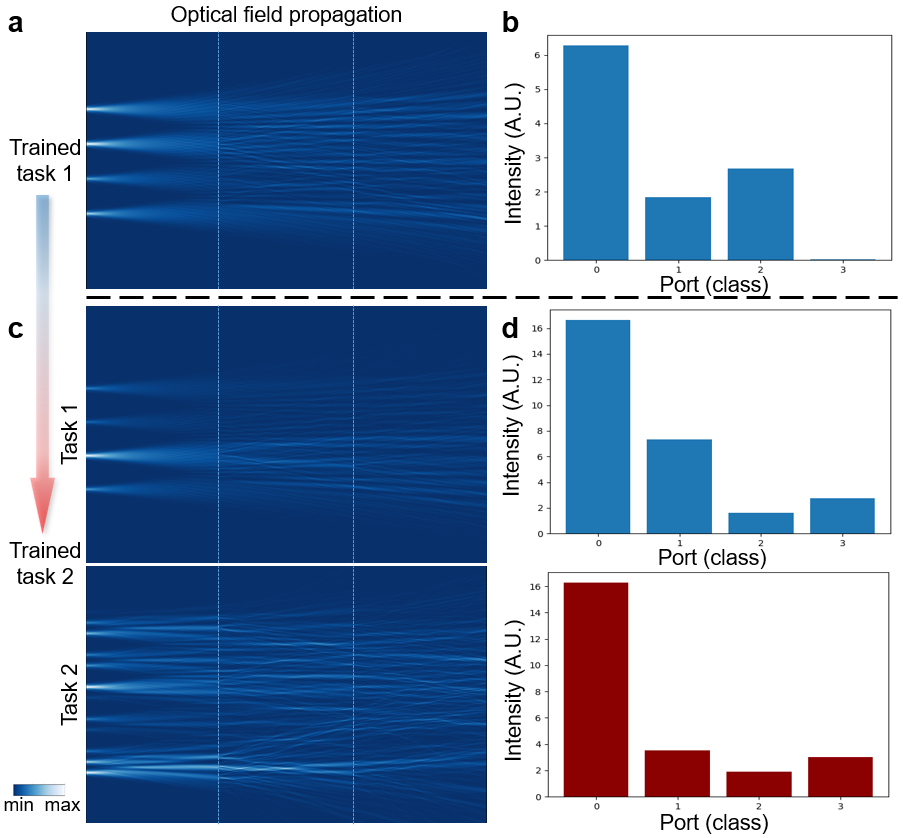


**Fig. S12: FDTD evaluations of on-chip L^2^ONN along with the lifelong learning process.** **a**, FDTD and **b**, light intensity of a testing sample after training task 1. The intensity detected at 4 output ports delivers the final classification result. **c**, FDTD and **d**, light intensity of 2 inference samples after training task 2, the chip successfully running both tasks. The sparsely activated photonic neurons are incrementally etched on slots to implement all learned tasks.


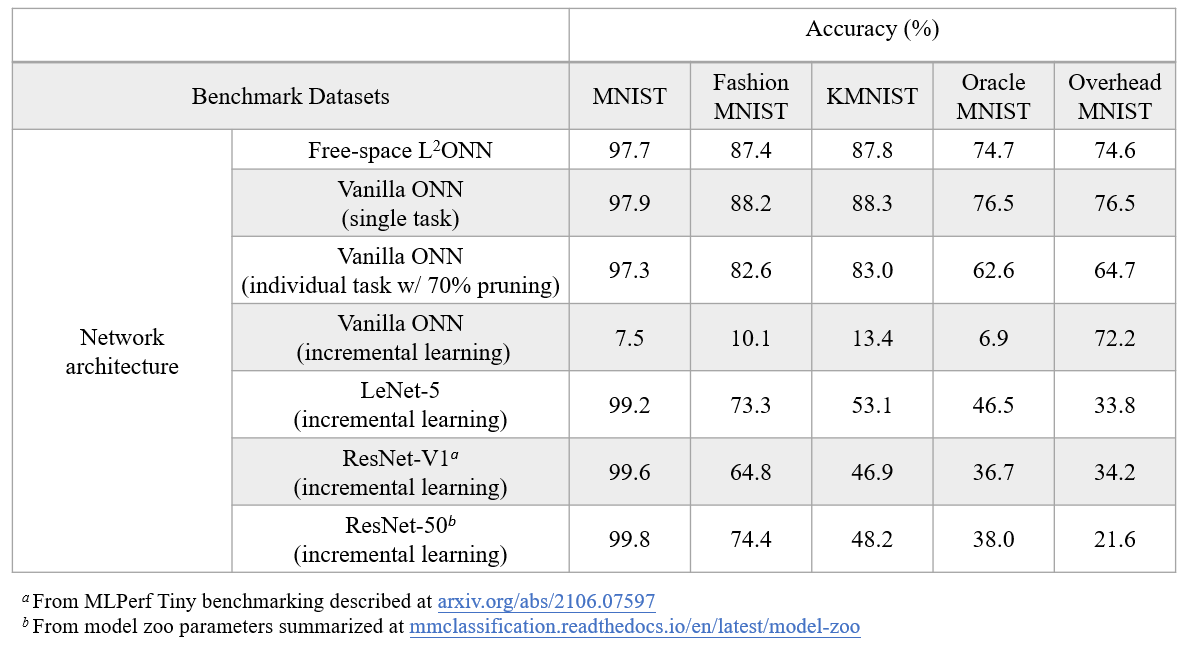


**Table S1: Accuracy summarization of different benchmarks on 5 representative vision classification tasks.**


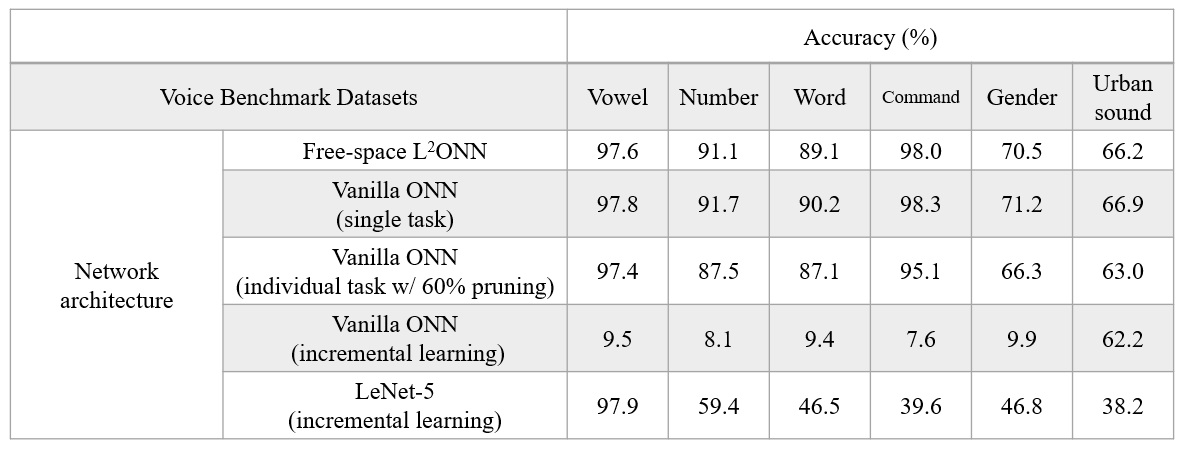


**Table S2: Accuracy summarization of different benchmarks on 6 voice recognition tasks.**


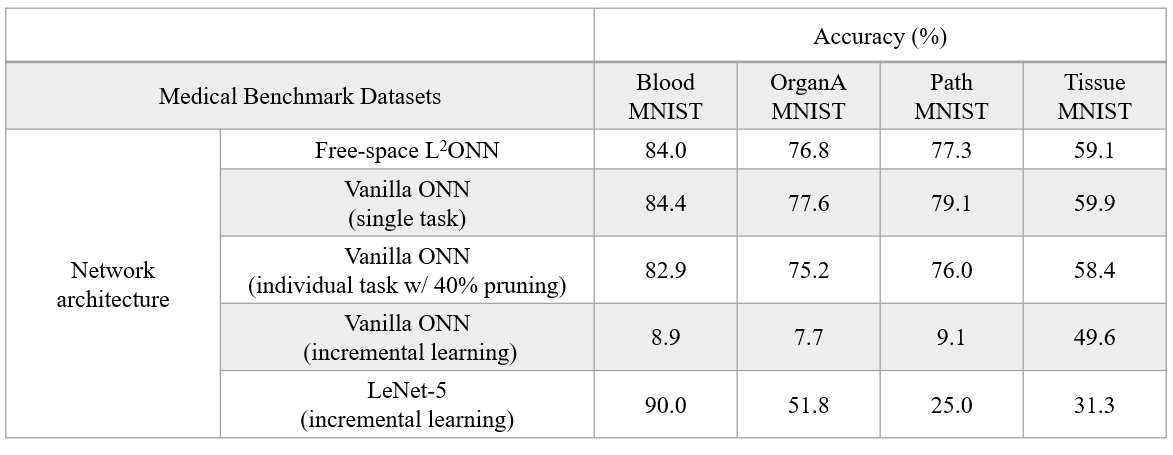


**Table S3: Accuracy summarization of different benchmarks on 4 medical diagnosis tasks.**


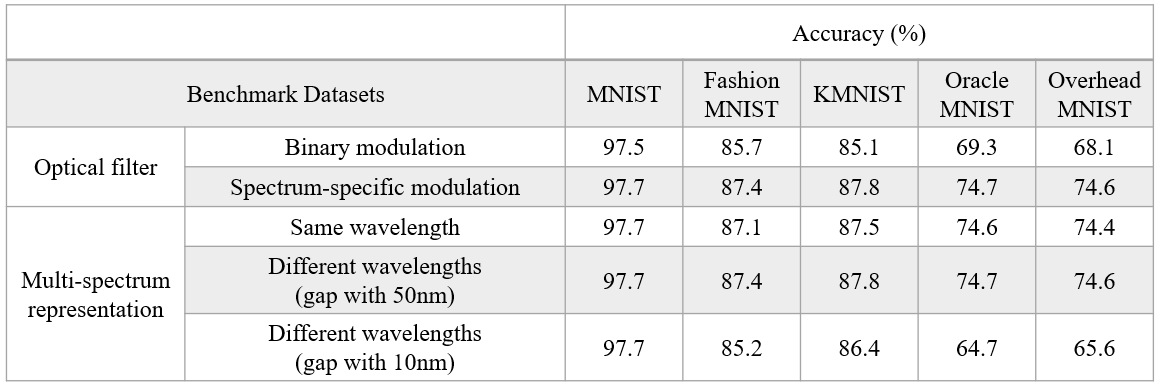


**Table S4: Ablation analysis of accuracy on optical filter and multi-spectrum representation.**

**Note S1:** **Computing efficiency analysis.**

In the free-space implementation, L^2^ONN is composed of 3 sparse optical convolution layers with optical filters and phase modulators, where each consists of 3 instant light-switching processes, 3 diffractive propagations, 3 complex field summations, and 1 intensity activation. Specifically, the optical energy source was powered by a multi-spectrum laser (TSL-570, Santec [1]). Spatial light modulators (SLMs, Meadowlark [2]) were employed for feature propagation and optical modulation. A scientific CMOS (sCMOS, Andor Zyla [3]) sensor was applied for detector at the output plane. To ensure synchronization and high-parallelism processing, the frame updates of the laser, SLMs, and sCMOS sensor were coordinated using external triggers to manage the massive data flow.

As noted in [4], for spatial resolution $R$ by $R$, each diffractive propagation takes $R^{2}\times(4\times R^{2}-1)= 4\times R^{4}-R^{2}$ real operations, and both phase modulation and intensity activation take $6\times R^{2}$ real operations. Hence each layer takes $2\times\left（ 4\times R^{4}-R^{2} \right）+2\times6\times R^{2}=8\times R^{4}+10\times R^{2}$ real operations. It indicates that there need$24\times R^{4}+30\times R^{2}$ real operations for optical convolutional module. Given that the maximal spatial resolution is 800×800 ($R$ = 800), L^2^ONN will relate to 9.83 TOPs (real operations) in total.

The inference speed of L^2^ONN is mainly bounded by the frame rates of SLMs and sensors. The updating speed of SLM could reach 1,436 FPS and the applied sensor supports ~145 FPS. Hence, the inference speed of each layer could reach 0.0068 s per frame (145 FPS). The total inference time would be 0.0068×3 = 0.0204 s, leading to a computing power of 9.83 TOPs / 0.0204 s = **481.863 TOPs s^-1^**. Note that it is promising to significantly promote the system speed by replacing the sensor to an ultrafast one (e.g., XXRapidFrame from Standford Computer Optics supports 100 billion FPS [5]).

In terms of power consumption, only laser sources, SLMs and sensors need power supplies and their powers are 10 Watts, 20 Watts, and 25 Watts, respectively. Note that during the inference process, the optical filters are fixed without any cost of light switching energy. The whole system needs (10+20+25) ×0.0204=1.122 J, leading to an energy efficiency of 9.83 TOPs / 1.122 J =**8.76 TOPs J^-1^**. Comparatively, for an NVIDIA GTX 1080 Ti GPU at maximum power, the computing capacity is **11.34 TOPs s^-1^** (float-point operations) and the power is 250 Watts TDP (**0.045 TOPs J^-1^**).

[1] <https://www.santec.com/products/instruments/tunablelaser/tsl-570/>

[2] <https://www.meadowlark.com/1024-x-1024-slm/>

[3] <https://andor.oxinst.com/products/scmos-camera-series/zyla-4-2-scmos>

[4] Zhou, T., Lin, X., Wu, J., Chen, Y., Xie, H., Li, Y., ... & Dai, Q. (2021). Large-scale neuromorphic optoelectronic computing with a reconfigurable diffractive processing unit. *Nature Photonics, 15*(5), 367-373.

[5] <https://stanfordcomputeroptics.com/products/iccd-framing-camera.html>

**Note S2:** **On-chip fabrication.**

The whole on-chip L^2^ONN architecture consists of a 4$\times$4 channels data-input grating coupler array, a dual-layer modulation area and a 4$\times$1 channels results read-out grating coupler array. Specifically, the chip is fabricated with $220 nm$ silicon waveguide SOI process, where the width of each waveguide is set as $450 nm$. The wedge shape in each input/read-out port and the layout of each grating coupler are simulated with finite-difference time-domain (FTDT) solver for the optimized energy coupling efficiency. In the modulation area, each layer contains $1,000$ stand-alone photonic neurons with a total width of $300 \mu m$, each neuron shares the identical width of $300 nm$, and the length is designed and etched with training error-propagation. The distances between layers, input ports and read-out ports are fixed as $50 \mu m$.

When deploying the lifelong learning properties on chip, the photonic neurons are incrementally etched to the design region task by task, which depends on the training process as illustrated in Fig. 5b.
